# Host–gut microbiota interactions during pregnancy

**DOI:** 10.1093/emph/eoae001

**Published:** 2024-01-06

**Authors:** Katherine R Amato, Priyanka Pradhan, Elizabeth K Mallott, Wesley Shirola, Amy Lu

**Affiliations:** Department of Anthropology, Northwestern University, Evanston, IL 60208, USA; Feinberg School of Medicine, Northwestern University, Chicago, IL 60611, USA; Department of Anthropology, Northwestern University, Evanston, IL 60208, USA; Department of Biology, Washington University in St. Louis, St. Louis, MO 63130, USA; Department of Psychology, Northwestern University, Evanston, IL 60208, USA; Department of Anthropology, Stony Brook University, Stony Brook, NY 11794, USA

**Keywords:** pregnancy, gut microbiota, hormone, metabolism, immunity, maternalfetal

## Abstract

Mammalian pregnancy is characterized by a well-known suite of physiological changes that support fetal growth and development, thereby positively affecting both maternal and offspring fitness. However, mothers also experience trade-offs between current and future maternal reproductive success, and maternal responses to these trade-offs can result in mother–offspring fitness conflicts. Knowledge of the mechanisms through which these trade-offs operate, as well as the contexts in which they operate, is critical for understanding the evolution of reproduction. Historically, hormonal changes during pregnancy have been thought to play a pivotal role in these conflicts since they directly and indirectly influence maternal metabolism, immunity, fetal growth and other aspects of offspring development. However, recent research suggests that gut microbiota may also play an important role. Here, we create a foundation for exploring this role by constructing a mechanistic model linking changes in maternal hormones, immunity and metabolism during pregnancy to changes in the gut microbiota. We posit that marked changes in hormones alter maternal gut microbiome composition and function both directly and indirectly via impacts on the immune system. The gut microbiota then feeds back to influence maternal immunity and metabolism. We posit that these dynamics are likely to be involved in mediating maternal and offspring fitness as well as trade-offs in different aspects of maternal and offspring health and fitness during pregnancy. We also predict that the interactions we describe are likely to vary across populations in response to maternal environments. Moving forward, empirical studies that combine microbial functional data and maternal physiological data with health and fitness outcomes for both mothers and infants will allow us to test the evolutionary and fitness implications of the gestational microbiota, enriching our understanding of the ecology and evolution of reproductive physiology.

## INTRODUCTION

Pregnancy is a critical period that relies on a delicate balance between maternal and fetal physiological requirements [1]. Mothers must provide the developing fetus with sufficient nutritional resources while simultaneously meeting their own nutritional requirements. They must also downregulate the immune system to protect the fetus from rejection while maintaining the ability to fight off pathogens that could contribute to maternal morbidity. Achieving these physiological goals allows mothers to successfully navigate the evolutionary trade-offs between current and future reproduction and thereby optimize reproductive success. Although maternal and fetal interests are often aligned, the evolutionary optimum for each participant may differ. The resulting maternal–fetal conflict permeates all aspects of pregnancy [1, 2].

Hormonal changes during pregnancy are thought to play a pivotal role in these conflicts since they directly and indirectly influence maternal metabolism, immunity, fetal growth and other aspects of offspring development [3, 4]. Gestational hormones are derived from both maternal endocrine glands (e.g. the adrenal gland), as well as the placenta, which is derived partially from fetal tissue [5, 6]. Remarkably, the placenta also produces enzymes that filter maternally derived hormones so that fetal exposure to such hormones is mitigated [1]. Therefore, both mother and fetus can exert control over maternal physiology via hormone regulation. Understanding the mechanisms through which these hormones affect different physiological systems during gestation is essential for empirically studying maternal–fetal conflict.

While there is a rich history of studies examining how gestational hormones orchestrate dynamic physiological changes in metabolism and immunity to benefit maternal and/or fetal interests [3, 4], recent evidence implicates gestational hormones in shaping yet another major system: the maternal gut microbiota. A variety of studies have reported that the composition and function of the gut microbiome changes during pregnancy, and both observational and experimental studies have established associative, or even causal links between various hormones and gut microbial composition (Table 1). These patterns are not surprising given that, among other factors, most of the physiological traits that are known to shift during pregnancy—including hormones—interact with the gut microbiota in a bidirectional manner. For example, shifts in host immune function [32] and hormones [33] can alter the gut microbiota, which can then feed back to affect host metabolism [34], immunity [35] and behavior [36]. However, research mechanistically linking these systems during pregnancy is currently sparse, limiting our understanding of the most pivotal systems involved in this web. Further, most published papers exploring the gut microbiota during pregnancy are currently focused either on fetal/infant health outcomes or diseases such as pre-eclampsia and gestational diabetes [37–40]. The result is a limited understanding of the potential feedback between maternal physiology and the gut microbiota during a healthy pregnancy. Without this knowledge, it is difficult to investigate how changes in the gut microbiota contribute to maternal–fetal conflict since benefits to maternal or offspring fitness can vary even within a healthy pregnancy.

**Table 1.** Summary of current studies of the gut microbiota during healthy pregnancy (excel file)

Reference	Species name	Country	Data type	Design	Parity	Beta diversity change (effect size)	Alpha diversity change (direction)	Enriched taxa and functions	Depleted taxa and functions
**Pregnant vs non-pregnant**									
Gohir *et al.* [7]	Mouse (*Mus musculus*)	na	16S rRNA amplicon sequencing	Cross sectional pregnant and non-pregnant	Primiparous	Yes	Yes (decrease)	*Lactococcus, Bacteroides, Akkermansia*, *Bifidobacterium, Clostridium, Allobaculum, Bilophila, Prevotella, Moryella, Paenibacillus, Escherichia, Lachnobacterium, Turicibacter, Parabacteroides, Collinsella, Roseburia*	*Clostridium, Sarcina, Anaerotruncus, Coprobacillus*
Springer *et al.* [8]*	Verreaux’s sifaka (*Propithecus vearreauxi*)	Madagascar	16S rRNA amplicon sequencing	Irregular longitudinal sampling of cycling, pregnant, lactating	?	No	No	None	None
Elderman *et al.* [9]	Mouse (*Mus musculus*)	na	MITchip	Cross sectional pregnant and non-pregnant	na	na	na	*Allobaculum et rel*., *Lactobacillus salivarius et rel., Lactobacillus plantarum et rel., Unclassified Clostridiales XVI, Clostridium perfringens et rel., Lactobacillus paracasei et rel*., and *Roseburia intestinalis et rel*.	None
Mallott and Amato [10]*	White-faced capuchin (*Cebus capucinus*)	Costa Rica	16S rRNA amplicon sequencing	Irregular longitudinal sampling of cycling, pregnant, lactating	?	Yes	No	*Lachnospiraceae, Microbacteriaceae, Megasphaera, Stenotrophomonas, Roseomonas*	*Clostridiales, Collinsella*
Trosvik *et al.* [11]*	Gelada (*Theropithecus gelada*)	Ethiopia	16S rRNA amplicon sequencing	Two-time point sampling of cycling, pregnant, lactating	?	Yes (0.023)	?	*?*	?
Chen *et al.* [12]	Human (*Homo sapiens*)	China	16S rRNA amplicon sequencing	Third trimester vs postpartum	?	Yes	Yes (decrease then increase)	None	None
Antwis *et al.* [13]*	Eastern black rhinocerus (*Dicoris bicornis*)	na	16S rRNA amplicon sequencing	Irregular longitudinal sampling of cycling, pregnant, lactating	Primiparous vs multiparous	Yes (0.028)	?	*Aerococcaceae, Lachnospiraceae, Spirochaetaceae, Sporobacter, Succiniclasticum*	None
Sparvoli, *et al.* [14]	Human (*Homo sapiens*)	Brazil	16S rRNA amplicon sequencing	Cross sectional pregnant and non-pregnant	?	No	No	None	None
Mallott *et al.* [15]*	Phayre’s leaf monkeys (*Trachypithecus phayrei*)	Thailand	16S rRNA amplicon sequencing	Irregular longitudinal sampling of cycling, pregnant, lactating	?	Yes	Yes (decrease)	None	None
Sun *et al.* [16]*	Tibetan macaque (*Macaca thibetana*)	China	16S rRNA amplicon sequencing	Irregular longitudinal sampling of cycling, pregnant, lactating	?	Yes (0.13)	No	Anaeroplasmatales, Succinivibrionaceae, Anaeroplasmataceae, Aeromonadales, *Succinivibrio*	Bacteroidales, *Butyricicoccus, Catenibacterium*
Baniel *et al.* [17]*	Gelada (*Theropithecus gelada*)	Ethiopia	16S rRNA amplicon sequencing	Irregular longitudinal sampling of cycling, pregnant, lactating	?	No	No	Verrucomicrobiota, Epsilonbacteraeota *(Helicobacter)*	None
Shi *et al.* [18]	Tibetan antelope (*Pantholops hodgsonii*)	China	16S rRNA amplicon sequencing	Third trimester vs postpartum	?	Yes (0.031)	Yes (increase)	*Anaerofustis*, *Bacteroides*, *Coprococcus_2*, *Ruminiclostridium_5*, *Ruminococcaceae_UCG-007*	None
Rhoades *et al.* [19]	Rhesus macaque (*Macaca mulatta*)	na	16S rRNA amplicon sequencing, shotgun metagenomics	Third trimester vs postpartum	?	Yes	Yes (increase)	Alloprevotella, Actinobacillus, Anaerovibrio	Treponema, Lachnospiraceae, Methanosphaera
Webb *et al.* [20]	White-faced capuchin (*Cebus capucinus*)	Costa Rica	16S rRNA amplicon sequencing	Irregular longitudinal sampling of cycling, pregnant, lactating	?	No	No	Firmicutes	Epsilonbacteraeota
Across stages of pregnancy*									
Collado *et al.* [21]	Human (*Homo sapiens*)	Finland	qPCR	Second vs third trimester	?	na	na	*Staphylococcus aureus, Bifidobacterium*	*Bacteroides fragilis*
Santacruz *et al.* [22]	Human (*Homo sapiens*)	Spain	qPCR	Second vs third trimester	?	na	No	None	None
Koren *et al.* [23]	Human (*Homo sapiens*)	Finland	16S rRNA amplicon sequencing	First vs third trimester	55% first, 29% second	Yes	Yes (increase)	*Actinobacteria, Proteobacteria*, *Lactobacillus zeae, Clostridium perfringens, Streptococcus* sp., *Enterococcus faecalis, Propionibacterium* sp., *Clostridium ramosum, Streptococcus salivarius*	*Clostridium* sp., *Faecalibacterium* sp., Enterobacteraceae, Ruminococcaceae, *Ruminococcus bromii, Blautia* sp.*, Eubacterium rectale, Lachnospiraceae, Subdoligranulum*
Avershina *et al.* [24]	Human (*Homo sapiens*)	Norway	16S rRNA amplicon sequencing, qPCR	First vs third trimester	?	No	None	None	None
DiGiulio *et al.* [25]	Human (*Homo sapiens*)	USA	16S rRNA amplicon sequencing	Weekly throughout pregnancy	?	No	None	None	None
Jasarevic *et al*. [26]	Mouse (*Mus musculus*)	na	16S rRNA amplicon sequencing	Daily throughout pregnancy	?, first	Yes	Yes (decrease then increase)	Biosynthesis of amino acids and lipids	None
Goltsman *et al.* [27]	Human (*Homo sapiens*)	USA (same as DiGiulio)	Shotgun metagenomics	Weekly throughout pregnancy	?	No	Yes (decrease)	Acetate and lactate production	Iron biosynthesis
Smid *et al.* [28]	Human (*Homo sapiens*)	USA	16S rRNA amplicon sequencing	Second vs third trimester	?	?	Yes (increase)	Actinobacteria*, Actinomyces, Anaerococcus, Eggerthella, Facklamia, Finegoldia, Propionibacterium, Pseudomonas, Ralstonia, Shewanella, Tepidimonas*	None
Nuriel-Ohayon *et al.* [29]	Human (*Homo sapiens*)	Israel	16S rRNA amplicon sequencing	First vs third trimester	?	?	?	*Bifidobacterium*, *Neisseria*, *Blautia*, *Collinsella*	*Clostridium*, *Dehalobacterium*
Yang *et al.* [30]	Human (*Homo sapiens*)	China	16S rRNA amplicon sequencing, shotgun metagenomics	First, second, third trimester	?	Yes	No	None	None
Miller *et al.* [31]	Human (*Homo sapiens*)	USA (Hawaii)	16S rRNA amplicon sequencing	First, second, third trimester	?		Yes (decrease)	Enterobacteraceae	Lactobacillaceae, Prevotellaceae

**Figure 1.**
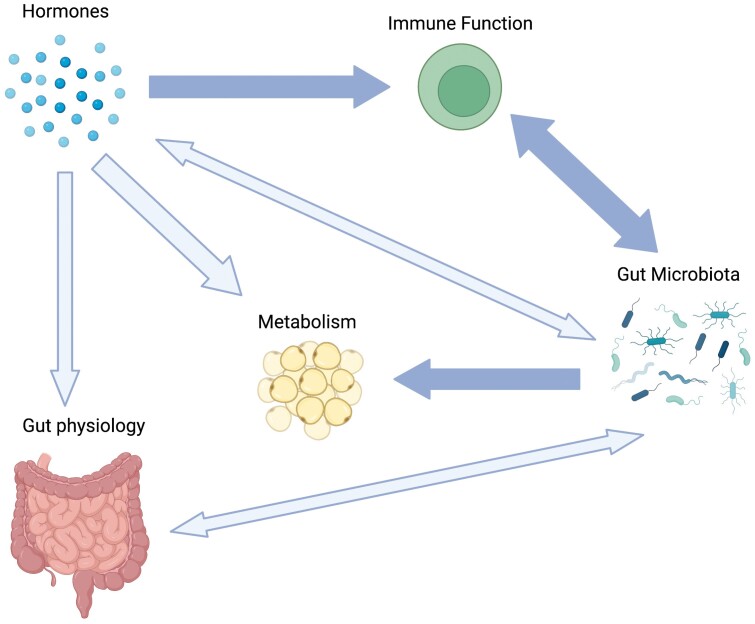
Model of host–microbe interactions during pregnancy. Darker arrows indicate proposed pathways likely to be most influential for maintaining a healthy pregnancy that involves the gut microbiota. Figure produced using BioRender.

To provide a foundation for exploring host–gut microbiota interactions during pregnancy from this evolutionary perspective, here we develop a putative model explicitly linking the gut microbiota to the physiological transformations experienced by mothers during pregnancy. We first posit that changes in gestational hormones are the primary driver of microbial changes during pregnancy, therefore establishing the gut microbiota as a pivotal system that can respond to maternal and fetal interests. To establish this mechanistic link, we take a deep dive into the bidirectional relationships between hormones, the gut microbiota, immunity and metabolism. We propose that marked changes in gestational hormones, including estrogens, progesterone and glucocorticoids (GCs) decrease maternal adaptive immune responses and increase innate immune responses, which then alters maternal gut microbiome composition and function. The gut microbiota then feeds back to influence maternal immunity and metabolism by regulating innate immune responses, glucose levels and lipid metabolism (Fig. 1). While physiological shifts in immunity, glucose regulation and metabolism could occur without microbial inputs, we theorize that microbial feedbacks provide functional redundancy, thus helping to mediate the balance between maternal and fetal fitness.

To create our model, we focus explicitly on host–microbe interactions. Although we acknowledge that various aspects of maternal physiology can affect each other directly, we cover this material briefly and direct interested readers to reviews on these topics for more in-depth information [3, 4, 41]. Additionally, we do not include changes in maternal behaviors, such as food intake, during pregnancy in our model. While these behaviors are likely to affect host–microbe interactions, our goal is to focus more narrowly on reproductive physiology (hormones, immunity, metabolism) and its interactions with the gut microbiota. Research in wild primates demonstrates that, while maternal diet can differ during pregnancy, variation in maternal gut microbiome composition is not associated with variation in maternal behaviors such as food intake and can, therefore, occur independently of diet change [10, 15, 20, 42]. Therefore, we aim to establish a model of these hormone–microbiota–physiology interactions before including the complexities of maternal behavior.

We continue by exploring the evolutionary and life-history implications of our model. If microbial shifts during pregnancy are driven both directly and indirectly by hormone shifts and affect maternal immunity and metabolism, they are likely a key system leveraged in maternal–fetal conflict. Identifying the magnitude of microbial contributions to maternal physiology and maternal and infant fitness will provide insight into the relative importance of the gut microbiota in the evolution of reproduction. For example, microbial contributions to maternal energy balance are likely to benefit both maternal and infant fitness during pregnancy but may also further benefit maternal fitness by facilitating a more rapid transition to the subsequent pregnancy. Similarly, microbial modulation of the immune system could benefit both maternal and infant fitnesses by increasing tolerance to the fetus but the gut microbiota may also further benefit maternal interests by actively excluding gut pathogens. Further, there are likely to be trade-offs in microbial functions themselves, specifically between regulation of immunity versus metabolism. Understanding how these dynamics intersect with maternal–fetal conflict as well as maternal environmental variation can provide mechanistic insight into health and fitness outcomes for both mother and infant.

Note that while we recognize that microbes are additional players in the evolutionary landscape, considering the conflicting interests of microbes dominant during gestation is outside the scope of this review. Given that all animals evolved in the presence of microbes, it is believed that their physiology has evolved at minimum to constrain negative microbial impacts and at maximum to exploit microbes to provide key functions [43]. These dynamics should operate during human pregnancy as well as outside of it. Our goal is to provide a foundation for testing the extent to which microbes contribute to maternal and/or infant fitness rather than identifying differences in host and microbial fitness interests.

## CHANGES IN MATERNAL PHYSIOLOGY DURING PREGNANCY

Pregnancy is characterized by major shifts in maternal biology, including changes in endocrine, cardiovascular, immunological, metabolic and neurological function. By and large, these changes have been framed in the context of evolutionary trade-offs: The damping of immunity and inflammation promote implantation and reduce fetal rejection; metabolic and cardiovascular shifts enhance available energy and the delivery of that energy to offspring and neurological changes prime the mother for maternal behaviors [44, 45]. However, these same processes weaken the maternal immune system, expose the mother to metabolic and cardiovascular disease and increase her likelihood of mental health complications in the postpartum period [44–46].

Hormonal changes initiated by both mother and fetus are driving many of these processes. Immediately following conception, fetal cells secrete human chorionic gonadotropin (hCG), maintaining the corpus luteum (CL)—a transient endocrine gland that usually recedes after each infertile cycle. The CL produces estrogen and progesterone during the first trimester, which together, contribute to the development and maintenance of the endometrium [47]. After the first trimester, the CL recedes, and the production of estrogens and progesterone is taken over by the placenta. At the same time, the pituitary gland secretes prolactin, which works synergistically with estrogen and progesterone to prepare the mammary gland for lactation [48]. While estrogens and progesterone peak during the third trimester and drop precipitously before birth, prolactin remains high throughout lactation, facilitating milk production. The pituitary also produces a substantial surge in oxytocin late in the third trimester, stimulating uterine contractions. Finally, maternal glucocorticoids (predominantly cortisol in humans) also increase during pregnancy, a process that is driven by the secretion of CRH (corticotropin-releasing hormone) from the placenta [48, 49]. As a catabolic hormone, increased cortisol during pregnancy frees up stored glucose, presumably to be used by the fetus, thus supporting some of the metabolic costs of pregnancy. Notably, several of these hormones—including prolactin, estrogens, progesterone and cortisol—also increase insulin resistance, a normal feature of pregnancy that increases glucose availability to the fetus and is thought to be driven mostly by placental lactogens [3, 50].

One of the key physiological systems that is altered by hormone shifts during pregnancy is the immune system. These changes arise from the unique problems posed by invasive placentation: a mother must prevent her own immune system from rejecting her fetus, while at the same time, maintaining some baseline level of immune function. For example, placental estrogen and progesterone dampen overall adaptive immune function by reducing the production of T cells [51, 52], presumably decreasing the chances of fetal rejection. Estrogen, progesterone and hCG also trigger the proliferation and enhancement of Treg cells [53–56], while hCG attracts them to the fetal–maternal interface [57] and regulates maturation of dendritic cells to promote tolerance [4, 58]. In addition, estrogen, progesterone, GCs and hCG inhibit the pro-inflammatory Th1 pathway by reducing pro-inflammatory cytokines and stimulate the anti-inflammatory Th2 pathway by increasing anti-inflammatory cytokines, particularly at the placenta interface [59–65]. More generally, GCs are critical in shutting down the immune response; however, reductions in GCs have been associated with autoimmune disease [66]. This latter example highlights the persistent parent–offspring conflict inherent in pregnancy: although maternal and fetal health and fitness are often synonymous, the physiological changes that enhance offspring fitness often come at a price to maternal fitness.

In addition to immunity, maternal metabolism also changes dramatically during pregnancy and is critical to maternal and fetal health. Early gestation is characterized by increased maternal fat stores and small increases in insulin sensitivity, allowing nutrients to be stored for future use during late gestation and lactation [67, 68]. Fat deposition during this period is substantial, with nonobese individuals gaining approximately 3.5 kg of subcutaneous fat across a normal pregnancy [69–71]. This period is characterized by increased leptin and other cytokines produced by maternal adipose tissue and the fetus, likely resulting in a diversion of nutrients to the fetus [72] and slight increases in insulin resistance [65]. By contrast, late gestation is characterized by exceeding high insulin resistance and fat utilization, which increases glucose and fatty acid availability to the fetus [67, 68]. As with other aspects of physiology in pregnancy, insulin insensitivity—which provides more energy to the fetus at the expense of maternal health—is driven by changes in placental hormones [73], including estrogens, progesterone and GCs [72, 74–77]. GCs, which are also known to mobilize energy, increase throughout pregnancy, suggesting that they also play an integral role in the shift from energy storage to energy mobilization during this time [78]. Such changes in maternal metabolism again reflect the competing interests of mother and offspring: although insulin resistance helps deliver more nutrients to the fetus, it can compromise maternal health by inducing metabolic dysregulation and gestational diabetes.

## EFFECTS OF MATERNAL PHYSIOLOGY ON THE GUT MICROBIOTA DURING PREGNANCY

Within the past 10 years, it has also become clear that maternal gut microbiome composition and/or function change during pregnancy, and may also play a role in maternal–fetal conflicts (Table 1). In 12 studies of humans [12, 14, 21–31] and 13 studies of non-human mammals [7, 9–11, 13, 15–20, 79] (Table 1) differences in microbial taxonomic composition and/or function have been identified across gestational stages (Table 1). However, which factors drive microbial shifts, and if and how those microbial shifts affect maternal physiology during pregnancy have not been directly investigated. The existing literature suggests that hormonal shifts likely contribute to concurrent changes in the maternal gut microbiota. While there are no paired hormone-microbiome data from humans, a longitudinal observational study of wild Phayre’s leaf monkeys, with an average of 5 samples per individual across 19 months [15], found a significant association between fecal progesterone levels and overall microbiome composition. Similarly, a longitudinal study of captive black rhinos with 4–12 samples per individual across 21 months [13] found a significant association between fecal progesterone, fecal GCs and the relative abundances of more than 50 microbial taxa. Two studies on wild nonhuman primates [80, 81] and one on pregnant wild squirrels [82] report associations between GCs and alterations in microbial diversity and composition, although the directionality of these relationships is not always consistent.

Observational studies cannot establish a causal role of hormones in microbial shifts during pregnancy, and there is evidence outside of pregnancy that the interactions between hormones and the gut microbiota are bidirectional [83, 84]. However, experimental work indicates that hormones do play a causal role in altering the gut microbiome, and these effects are generally larger than those of microbes on circulating hormone levels. For example, in non-pregnant individuals, experimentally delivered estrogen-like soy isoflavones alter the relative abundances of *Bifidobacterium* in both mice and postmenopausal humans [85, 86]. In addition, administration of 17B-estradiol to male and ovariectomized female mice results in a microbiome composition characteristic of an intact female [33]. Experimental studies on birds and fish show more conclusively that ecologically relevant increases in GCs alter microbial diversity [87–89]. One study on wild lizards provides some evidence that the directionality of these changes can be contingent on the female reproductive state [89]. Specifically, experimentally elevated GCs increased within-sample gut microbial diversity in late pregnancy, while the same treatment in non-pregnant females influenced gut microbial composition [89].

Although the causal pathway linking hormones to changes in the gut microbiome can be direct, hormones may also have indirect effects on the gestational gut microbiota by inducing changes in immunity. The immune system is known to carefully regulate the gut microbiota in non-pregnant individuals. Mucin secreted by host gut epithelial cells creates a physical barrier, while innate lymphoid cells maintain gut epithelial barrier function by repairing tissue damage and reducing inflammation [32, 90]. Antimicrobial peptides also kill microbes that overcome physical barriers in the gut [32, 90]. At the same time, the adaptive immune system monitors the gut microbiota via dendritic cells that traverse the mucin [91]. These cells can stimulate T and B cells in nearby tissue to produce cytokines and microbe-specific immunoglobulin A (IgA) to neutralize select microbes [32, 92]. As a result, variation in almost any aspect of the host immune system consistently leads to variation in the gut microbiota. It follows that hormone-induced changes in both the innate and adaptive immune system during pregnancy are likely to substantially alter the microbiota.

It is also theoretically possible that hormone-influenced shifts in maternal metabolism could alter the maternal microbiota. However, while abundant data show an association between host metabolism and the gut microbiome, the causal direction is primarily gut microbiome-to-metabolism rather than vice versa. There is a dearth of studies testing how host metabolism affects the gut microbiome. Therefore, the current literature suggests that maternal microbial shifts are driven directly by hormone fluctuations during pregnancy, as well as by the effects of hormone fluctuations on the maternal immune system (Fig. 1). Future research could alter this interpretation.

## EFFECTS OF THE GUT MICROBIOTA ON MATERNAL IMMUNITY AND METABOLISM DURING PREGNANCY

Changes in the gut microbiota triggered either directly or indirectly by maternal hormone fluctuations are likely to feed back to affect maternal physiology. Studies outside the context of pregnancy have demonstrated that gut microbiota can stimulate the innate immune system by triggering pro-inflammatory cytokine production in gut epithelial and immune cells with microbe-associated molecular patterns [93]. At the same time, the microbiota can regulate adaptive immunity by promoting anti-inflammatory Th2 pathways [93]. For example, many microbial taxa (e.g. Proteobacteria, *Lactobacillus, Clostridium, Bacteroides fragilis, Eubacterium hallii, Faecalibacterium prauznitzii* and *Roseburia intestinalis*) can induce expression of Treg cells and production of anti-inflammatory cytokines [69, 71, 94–97]. Additionally, microbial production of short-chain fatty acids (SCFAs), such as acetate and butyrate, can induce anti-inflammatory cytokine production and Treg expression [98, 99]. Butyrate can also improve epithelial barrier integrity in the gut, limiting immune system exposure to microbes and subsequent inflammatory responses [100]. These types of microbial processes that dampen the adaptive immune system while regulating the innate immune response could help pregnant individuals maintain their altered immune state, providing redundancy in the system. Although few studies have directly tested this hypothesis, germ-free mice undergo fewer immune changes during pregnancy compared to conventional mice. Specifically, they do not show increased Treg or innate immune responses [101]. These data suggest that microbial changes play a role in supporting and maintaining the changes in the innate and adaptive immune system characteristic of a healthy pregnancy.

Changes in the gut microbiota during pregnancy are also likely to affect maternal metabolism [102]. Experiments with non-pregnant germ-free mice have demonstrated that the gut microbiota can causally incite fat deposition in hosts [103, 104]. There are multiple pathways through which this can occur. First, changes in the amounts and types of SCFAs produced by the gut microbiota can promote fat deposition [105]. For example, butyrate inhibits adipogenesis while acetate incites it [103, 106, 107]. SCFAs can influence fat deposition directly, by acting as additional energy sources, and indirectly, by modifying hormone concentrations, neurotransmitter levels and gene expression patterns [108–111]. Beyond SCFAs, microbial metabolism of choline and bile acids can affect host energy and lipid metabolism [7, 110], and decreases in serotonin production by the gut microbiota lead to impaired host lipid metabolism [112, 113]. Variation in microbial metabolites is also associated with altered insulin regulation in non-pregnant humans and mice [103, 106, 107]. For example, the SCFA propionate promotes gluconeogenesis [114, 115], and a deficiency in serotonin production by the microbiota leads to impaired host glucose regulation [112, 113].

These microbial pathways could play an important complementary role to maternal hormones in regulating metabolism during pregnancy. In fact, a study of germ-free mice colonized with gut microbes from humans in either their first or third trimesters of pregnancy found significant adipose tissue accumulation in mice that received the third-trimester microbiota, indicating that the microbiota is likely to have a causal effect on maternal metabolism during pregnancy [23]. No other studies have directly assessed microbial impacts on maternal metabolism during pregnancy. However, research in non-pregnant individuals indicates that host immune dynamics can affect the gut microbiota in ways that ultimately alter host metabolism. For example, Treg activity in mice is positively associated with microbial energy harvest and host adiposity [116]. Because Treg activity increases during pregnancy [51], the maternal microbiota is likely to shift in a way that encourages fat deposition.

## TOWARD A MODEL OF HOST–MICROBE INTERACTIONS DURING PREGNANCY

Based on the current state of the literature, and the central role of hormones as upstream regulators of physiological function, we posit that hormones are the primary drivers of gut microbial shifts during pregnancy and that these microbial shifts help maintain and support changes in maternal metabolism and immunity commonly associated with successful pregnancy (Fig. 1). Hormones can accomplish this via two pathways: First, some placental reproductive hormones—such as estrogen—can influence the maternal gut microbiota indirectly, via impacts on the maternal immune system. Second, direct effects of placental hormones on the maternal gut microbiota are also possible. Although some experimental studies in mice suggest that estradiol injections can directly alter the gut microbiome, experimental elevations in estradiol may not mimic rises in estrogens during pregnancy, which are dominated by estrone, rather than estradiol [117]. However, because the predominant form of GCs (i.e. cortisol in humans) does not change during pregnancy, experimental studies on GCs in animal models better mimic natural GC elevations [87–89], suggesting that GC effects on the microbiota may be more direct and potent. Importantly, maternal GCs are embedded in a feedback loop that implicates the involvement of both maternal and fetal interests: in humans, maternal cortisol production causes an increase in placental CRH, which further drives maternal cortisol production (Fig. 1). Thus, we posit that hormones of maternal and placental origin are primarily responsible for the changes in the gestational gut microbiota, which then feeds back to regulate maternal immune and metabolic phenotypes during pregnancy (Fig. 1). These dynamics would make the gut microbiome an additional mediator of maternal–fetal conflict.

We recognize that endocrine changes and other mechanisms can incite adaptive physiological changes in immunity and metabolism during pregnancy, independently of microbial inputs. The ability of germ-free animals to successfully reproduce is clear evidence of this fact. However, because bacteria predate animals evolutionarily, all animals have evolved to interact with microbes [43]. In some cases, these interactions involve animal adaptations to avoid or exclude microbes, but in others, they are likely to involve animals exploiting microbial functions to replace or complement their own genetically encoded functions (e.g. fiber and lactose digestion, respectively [118]). As a result, we hypothesize that the gut microbiome provides functional redundancy during pregnancy, shifting in response to changes in maternal physiology and feeding back to reinforce many of the same changes. This redundancy could have positive fitness effects for both mother and fetus.

Ultimately, studies will need to pair microbial and host physiological data with measures of host fitness to examine the evolutionary implications of gestational changes in the gut microbiota. At a foundational level, we must determine whether the magnitude of microbial impact on maternal immunity and metabolism is enough to affect pregnancy outcomes. More specifically, we must identify whether microbial influences on maternal physiology benefit maternal fitness, offspring fitness or both. Maternal physiological changes such as increased insulin insensitivity and downregulation of adaptive immunity clearly benefit fetal health by improving maternal ability to carry offspring to term, have successful births, and give birth to larger offspring, which contributes to fetal fitness.

In most cases, increased fetal fitness contributes to increased maternal fitness. However, maternal–fetal interests are not always consistently aligned [1, 2, 119]. For example, mothers should constrain offspring energy utilization both before and after birth to ensure that maternal body condition will support future pregnancies and their offspring. It is unclear whether fetally driven changes in the gut microbiome influence these parent–offspring dynamics by altering overall energy availability or influencing maternal metabolism. Because the endocrine functions of the placenta are located primarily at the synctiotrophoblast, which is part of the fetal compartment, the involvement of gestational hormones as regulators of gut microbial shifts suggests that fetuses may be driving many of these changes. Fetuses, therefore, may also obtain the primary fitness benefits. Notably, gestational stress may represent a more complicated situation where maternal GCs can increase due to direct stimulation of the maternal HPA axis (via ‘stress’), or it can be stimulated by the fetus, via placental CRH secretion [120]. Because of these complicated dynamics, adaptive hypotheses of microbial changes during pregnancy must be tested by measuring microbiome function in relation to both maternal and offspring fitness. For instance, if microbial shifts result in faster resumption of maternal fertility, but not increased neonatal body mass, we could conclude that the benefits are largely reaped by the mother, not the infant.

Beyond focusing solely on metabolism, we must also understand how microbial impacts on the immune system interact with these physiological trade-offs and conflicts during pregnancy. Microbial taxa that promote host energy acquisition are not necessarily the same taxa that provide hosts with immune benefits or anti-inflammatory functions. Outside of pregnancy, microbes that promote host digestive efficiency and energy metabolism may also promote inflammation or reduce protection against pathogens [121, 122]. Therefore, shifts in the microbiota that favor a higher maternal capacity for energy acquisition and immuno-modulation to maintain fetal growth could occur at the expense of facilitating a pro-inflammatory milieu or reducing defenses against infection. Some indirect evidence of these dynamics exists in the literature. The maternal gut microbiota has an increased abundance of pro-inflammatory microbial taxa during pregnancy, including Actinobacteria and Firmicutes, as well as decreased abundance of anti-inflammatory taxa, such as Bifidobacterium and Faecalibacterium [22]. It has also been suggested that mucin-degraders such as *Akkermansia*, which are reported to be enriched during pregnancy, allow enhanced energy extraction at the cost of decreasing the integrity of the gut epithelial barriers [21, 7, 123]. The increased ‘leakiness’ of the gut may be a tradeoff, allowing the mother to absorb more vitamins and nutrients for the growth of the fetus at the cost of increased infections [73, 124]. However, more research is necessary to fully understand gut barrier integrity during pregnancy given the beneficial effects of estradiol and butyrate-producing microbes that are often enriched [125].

Microbial shifts during pregnancy may also contribute to maternal and infant fitness by affecting post-birth infant development. For example, lactation increases maternal daily energy expenditure in humans by nearly 33% [126], and these demands can affect both infant outcomes and maternal ability to conceive again [127, 128]. Microbial contributions to maternal energy stores before and during this period could, therefore, improve both maternal and infant fitness. In addition, infant guts are thought to be sterile at birth, with the first ‘inoculation’ occurring as infants are exposed to maternal microbes during the process of birth. These microbes originate largely not only from the maternal vaginal and gut microbiome as a result of infant contact with the maternal urogenital area but also from skin [129, 130]. As a result, while shifts in the maternal gut microbiota during pregnancy may have important physiological effects for both the mother and the fetus, changes in the third trimester also influence the types of microbes infants are likely to be exposed to. These initial exposures are likely to shape the developmental trajectory of the infant microbiota [129, 131], ultimately affecting infant physiology and health outcomes. For example, the maternal fecal microbiome predicts gestational age at birth, birth weight and neonatal growth in rural Zimbabwe [132]. Specifically, resistant starch degradation and vitamin B metabolism predicted increased birth weight and neonatal growth, while biofilm formation predicted reduced birth weight and growth. Additionally, infant immune responses to pathogens are likely to be dictated by these early-life microbial exposures. Not only can microbes actively exclude pathogenic microbes from host bodies, but they actively train the immune system during early life [133–135]. Therefore, microbial impacts on maternal and offspring fitness go beyond metabolic benefits to include reduced infant morbidity and mortality in the face of infectious disease.

Importantly, the physiological dynamics and trade-offs discussed above are likely to vary in response to host ecology given the plasticity of both the placenta and the microbiome to environmental variation. To begin with, abundant evidence shows that the placenta fine-tunes fetal nutrition and development in response to environmental cues filtered from the mother [136]. Thus estrogens, progesterone and CRH secretion are expected to adaptively integrate cues from the mother to influence fetal health. Studies in humans and other animals, for example, show that proxies of maternal condition, such as parity and age [137–139], or even environmental variables, such as rainfall, can influence the pattern of estrogen, progesterone and GC secretion across pregnancy. Given these effects, we might also expect maternal microbial shifts to respond similarly [140]. However, the precise beneficiary of such shifts is not a straightforward prediction. Studies on humans and nonhuman primates suggest that mothers could prioritize future reproduction over offspring health, in both extremely poor and rich conditions [119]. Thus, while extrinsic and intrinsic predictors of maternal condition are expected to influence microbial responses, the beneficiaries of such responses must also be examined.

Differential access to nutrition may also influence how mothers navigate microbially driven tradeoffs between metabolism and immunity. For example, females that are more energetically constrained may prioritize metabolic shifts over immune shifts, leading to a gut microbiome profile that is biased toward short-chain fatty acid producers in families such as Lachnospiraceae and Ruminococcaceae. Recent research on pigs, for example, suggests that first-time mothers who must fuel their own growth while also supporting the energetic costs of reproduction are characterized by a slower, different pattern of gut microbiome remodeling during pregnancy compared to multiparous females [140]. However, few other studies to date have investigated the role of parity in the gestational microbiome (Table 1). Furthermore, numerous other life history, ecological and social factors such as maternal age, infant sex, rainfall, exposure to hypoxia (altitude) and maternal social or socioeconomic status may influence ‘microbial allocation decisions’ to tilt toward larger or smaller investments in metabolism over immunity. Many of these factors are known to alter endocrine and other aspects of placental physiology [136], suggesting that the fitness implications of gut microbial remodeling may be better understood by examining individual responses to these challenges.

In a similar way, ecological conditions related to disease exposure may also alter the magnitude of gut microbiome remodeling during pregnancy. For instance, pregnant women living in environments with increased exposure to parasites and bacterial pathogens appear to undergo less pronounced immune shifts during pregnancy since their immune systems are already challenged by pathogens that elicit strong innate immune responses [141]. These dampened immune shifts could lead to dampened microbiota shifts. This example again highlights how integrating measures of host physiology will allow us to better identify the conditions that elicit or constrain changes in the microbiota, allowing us to better understand when such changes could be the most critical to fetal or maternal health and fitness.

## POTENTIAL MEDICAL APPLICATIONS

As implied above, increased consideration of the role of the gut microbiota in shaping overall maternal physiology during pregnancy, as well as the dynamics involved in potential maternal physiological trade-offs could improve understanding of gestational disease states and poor pregnancy outcomes. For example, an over-representation of microbial taxa or genes that increase energy production and alter maternal metabolism could lead to reduced immunity and pathologies such as increased viral, bacterial, and parasitic infections, in addition to excessive weight gain [21]. Similarly, other negative pregnancy outcomes including gestational diabetes, pre-eclampsia, intrauterine growth restriction, and miscarriage have been suggested to correlate with differences in the gut microbiome [73, 142, 143]. While there are no studies yet that causally link variation in the maternal microbiome with the development of pregnancy complications, the associations of microbial variation with other diseases and negative health outcomes suggest that similar relationships are likely to exist during pregnancy [142, 143]. Given concerns about pharmaceutical use during pregnancy, microbial interventions and therapies to avoid common negative health outcomes during pregnancy would be a valuable tool [144].

Nevertheless, we need more information about maternal-microbe dynamics during pregnancy before we can develop these types of treatments. Changes in the gut microbiota can be deleterious or beneficial depending on context. For example, shifts in the gut microbiota that promote increases in adiposity to allow for fetal growth during pregnancy may increase the risk of metabolic disorders in non-pregnant individuals [23]. Similarly, short-term increases in inflammation as a result of increasing microbially mediated lipid metabolism may be beneficial if the improvement in maternal and fetal nutrition and health outweighs the deleterious consequences of increased inflammation over a sustained period of time. While this trade-off may be essential for all pregnancies, it may be more important for mothers who are energetically stressed prior to or during pregnancy. Similarly, the pathogen landscape that mothers are exposed to may alter their immune needs during pregnancy [141], which is tightly linked to the microbiota and its subsequent influence on other body systems. Therefore, the context of pregnancy, in general, as well as the specific environmental variables acting on any single pregnancy, must be accounted for before we begin to consider altering microbiota.

## CONCLUSIONS

In conclusion, the gut microbiota is likely to play a critical but understudied role in the physiological shifts associated with pregnancy. Based on our current understanding of the literature, we propose that changes in hormones during pregnancy trigger changes in the maternal immune system, which then alters the gut microbiota. The gut microbiota then feeds back to maintain altered maternal immunity and metabolism. We hypothesize that these dynamics contribute to maternal and offspring fitness and may be important for understanding maternal–offspring conflict both during and after pregnancy. They may also be involved in mediating trade-offs in maternal metabolic and immune phenotypes during pregnancy. It is likely that the magnitude of interactions and their impacts on maternal and offspring fitness vary across populations in response to maternal environments and associated biology. A careful exploration of these processes could help shape future research into the maternal microbiota and pregnancy outcomes and may eventually lead to clinical decision-making that is based on a more complete understanding of the complex changes during pregnancy.

To achieve a more complete understanding of microbial contributions to pregnancy phenotypes, there are a range of questions that must be addressed empirically. For example, to what extent are microbes affecting maternal physiology during pregnancy? Fetal growth? How much of an impact do these effects have on maternal and offspring fitness? Which members of the gut microbiota are most responsible for these impacts? Can social and ecological factors encountered by pregnant individuals disrupt these host–microbe interactions? Which ones and how easily? These questions are not easily addressed with the current literature. However, they are critical to understanding how the gut microbiota interacts with maternal physiological processes during pregnancy and are likely to provide key insight into maternal and fetal health outcomes.

To begin to address these questions, studies are needed that fully characterize the taxonomic and functional changes that occur throughout a healthy pregnancy in multiple populations and while simultaneously characterizing the effects of diet, social and physical environments and health conditions on these patterns. Mechanistic studies should also investigate the relationship between hormones and the gut microbiota more fully, as well as the complex bidirectional interactions between the gut microbiota and both maternal metabolism and immune function/inflammation during pregnancy. While some of these topics are currently being investigated, many focus on disease states and poor maternal and/or infant outcomes [145, 146]. An improved understanding of microbial dynamics and the effects on maternal and fetal physiology in healthy pregnancies will not only better inform our approach to disease but will also provide key insight into the evolutionary role of the human microbiota during this sensitive period.
